# Chemical Factory‐Guaranteed Enhanced Chemodynamic Therapy for Orthotopic Liver Cancer

**DOI:** 10.1002/advs.202201232

**Published:** 2022-06-16

**Authors:** Zhongmin Tang, Shiman Wu, Peiran Zhao, Han Wang, Dalong Ni, Huiyan Li, Xingwu Jiang, Yelin Wu, Yun Meng, Zhenwei Yao, Weibo Cai, Wenbo Bu

**Affiliations:** ^1^ Tongji University Cancer Center Shanghai Tenth People's Hospital Tongji University School of Medicine Shanghai 200072 P. R. China; ^2^ Departments of Radiology, Medical Physics, Materials Science & Engineering Pharmaceutical Sciences University of Wisconsin − Madison Madison WI 53705 USA; ^3^ Department of Radiology Huashan Hospital Fudan University Shanghai 200040 P. R. China; ^4^ Department of Materials Science and State Key Laboratory of Molecular Engineering of Polymers Fudan University 220 Handan Road Shanghai 200438 P. R. China; ^5^ Ruijin Hospital Shanghai Jiao Tong University School of Medicine Shanghai 200240 P. R. China

**Keywords:** chemical factory, Fenton, liver cancer, metabolism, zinc peroxide

## Abstract

In the field of nanomedicine, there is a tendency of matching designed nanomaterials with a suitable type of orthotopic cancer model, not just a casual subcutaneous one. Under this condition, knowing the specific features of the chosen cancer model is the priority, then introducing a proper therapy strategy using designed nanomaterials. Here, the Fenton chemistry is combined with zinc peroxide nanoparticles in the treatment of orthotopic liver cancer which has a “chemical factory” including that liver is the main place for iron storage, metabolism, and also the main metabolic sites for the majority of ingested substances, guaranteeing customized and enhanced chemodynamic therapy and normal liver cells protection as well. The good results in vitro and in vivo can set an inspiring example for exploring and utilizing suitable nanomaterials in corresponding cancer models, ensuring well‐fitness of nanomaterials for disease and satisfactory therapeutic effect.

## Graphical abstract

The chemical factory of orthotopic liver cancer has laid a solid foundation for introducing chemodynamic therapy into orthotopic liver cancer. The orthotopic liver cancer therapy is realized through customized microenvironment/chemical factory‐guaranteed enhanced Fenton/Fenton‐like reactions by using the polyethylene glycol‐modified zinc peroxide (ZnO_2_@PEG) nanoparticles.

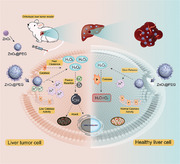

## 1 Introduction

The liver is a reasonably well‐equipped “chemical factory,” that entails a myriad of physiological processes.^[^ ^1^ ^]^ It maintains homeostasis of the whole body by regulating several diverse physiological functions,^[^ ^2^ ^]^ including substance synthesis, metabolic waste decomposition, and metabolism regulation.^[^ ^3^ ^]^ Carrying so many functions, it could be susceptible to negative stimulation, such as obesity and virus infection, and become diseased and malfunction, and in the worst case, develop tumors.^[^ ^4^ ^]^ Up to now, a lot of experimental therapies for liver cancer have been accomplished upon the liver subcutaneous tumor model^[^ ^5^ ^]^ rather than the orthoptic liver tumor model. An underlying shortcoming is that the subcutaneous tumor model cannot represent the actual physiological environment of human liver cancer. Thus, it is unreplaceable to introduce an orthoptic liver tumor model for the evaluation of designed therapeutic agents. The common characteristic of tumor, such as weak acidity,^[^ ^6^ ^]^ insufficient catalase activity, and intolerance of external reactive oxygen species (ROS),^[^ ^7^ ^]^ could be found in the orthoptic liver tumor model. Critically, the orthoptic liver tumor model's uniqueness lies in its origin, liver, the central place for iron storage,^[^ ^8^ ^]^ metabolism, and the primary metabolic sites for most ingested substances. Generally, these characteristics have laid a solid foundation for the introduction of chemodynamic therapy (CDT) strategy, which utilizes the Fenton/Fenton‐like reactions in vivo for cancer treatment. When compared with other ROS‐related therapy strategies such as photodynamic therapy, radiotherapy, and sonodynamic therapy, there is no external energy stimulation needed which could overcome penetration depth limitation. Moreover, all necessary parts for Fenton/Fenton‐like reactions could much enhance the specificity of CDT which could diminish potential side effects in healthy tissues.

Since the first proposal of CDT,^[^ ^9^ ^]^ this area has accomplished tremendous progress, yet few researches are focused on utilizing the specific environment of the orthotopic liver cancer model.^[^ ^10^ ^]^ There is a tendency of matching designed nanomaterials and the selected administration methods with a suitable type of orthotopic cancer model, not just a casual subcutaneous one, such as aerosol delivery of siRNA nanoparticles for orthotopic lung tumor therapy,^[^ ^11^ ^]^ or delivering the nebulized therapeutic mRNA to the lung^[^ ^12^ ^]^ which definitely can be used in orthotopic lung tumor therapy or pneumonia relief, etc. The orthotopic liver cancer model shares more characteristics with human liver cancer, guaranteeing the treatment efficiency and accelerating clinical translation. In general, the catalyst part of transition metals (like Fe and Cu) in most existing CDT research was provided by the components in the exogenous designed nanoparticles, neglecting the abundant amount of available iron inside the liver. Employing the iron‐enriched microenvironment in the liver could generate more H_2_O_2_ to kill the tumor. Ions with multiple functions, such as Ca^2+^, Fe^2+^, Mg^2+^, and Zn^2+^ not only serve as intracellular messengers, but also play a role in tumor growth, invasion, and metastasis.^[^ ^13^ ^]^ Interestingly, although a mild increase of Zn‐ion concentration is helpful against ROS elevation,^[^ ^14^ ^]^ cancer cells would downregulate or even silence the expression of zinc transporting proteins to avoid the antitumor effect of zinc itself.^[^ ^15^ ^]^ Typically, zinc is decreased in some cancer to avoid its tumor‐suppressing effects. The Zn^2+^ increase would coincide with the strategy of upregulating zinc levels for cancer therapy. Therefore, the coexistence of ROS with zinc accumulation could amplify the damaging effects on cancers. Thus, we hypothesized a rapid burst of zinc generated inside cancer cells could bypass the cancer cells’ zinc‐lacking shield and kill them by excessive ROS. With the above considered, the current problem that needs to be solved now is to find suitable nanomaterials that can specifically trigger the CDT via releasing H_2_O_2_ in orthotopic liver cancer and protecting surrounding healthy liver cells simultaneously.

**Figure 1:**
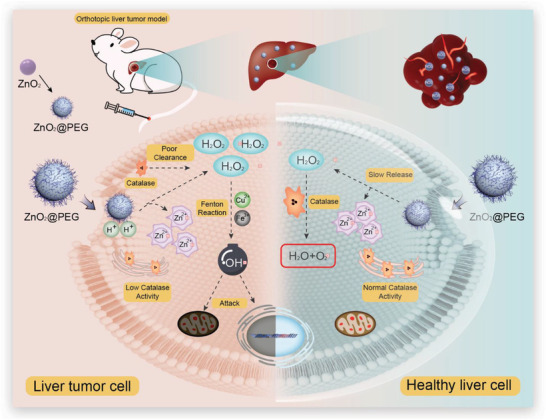
Schematic of chemical factory‐guaranteed enhanced CDT for orthotopic liver cancer.

In this work, we first established the orthotopic liver tumor model and realized the orthotopic liver cancer therapy through chemical factory‐guaranteed enhanced Fenton/Fenton‐like reactions by using the polyethylene glycol‐modified zinc peroxide nanoparticles (ZnO_2_@PEG NPs). As illustrated in **Figure** **1**, after being intravenously injected into mice, the ZnO_2_@PEG NPs could passively accumulate in the liver and liver cancer tissues. In tumor tissues, the ZnO_2_@PEG NPs would rapidly release Zn ion and H_2_O_2_ due to mild acidity and low catalase activity, subsequently triggering the enhanced Fenton/Fenton‐like reactions with the intrinsic Fe, utilizing hydroxyl radical and burst increased Zn ion for efficient therapy. In contrast to nontumor liver tissues, the production of Zn ion and H_2_O_2_ is prolonged and the H_2_O_2_ will be soon degraded into water and oxygen, attributed to the physiologically existed catalase inside the regular cells of the liver. Besides, the mild increase of Zn‐ion concentration strengthens the anti‐ROS ability of healthy liver cells to prevent potential side effects. Moreover, the ZnO_2_@PEG NPs in tumor tissues would inhibit the orthotopic liver cancer metastasis to some degree, which is presented in our in vivo results. In general, inspired by the characteristic of orthotopic liver cancer microenvironment including mild acidity, low catalase activity, high Fe concentration, and nanomaterials enrichment, we tailored nanomaterials and realized specific rapid release of H_2_O_2_ for triggering enhanced Fenton/Fenton‐like reactions, collectively inducing efficient orthotopic liver cancer therapy.

## 2 Results and Discussion

**Figure 2:**
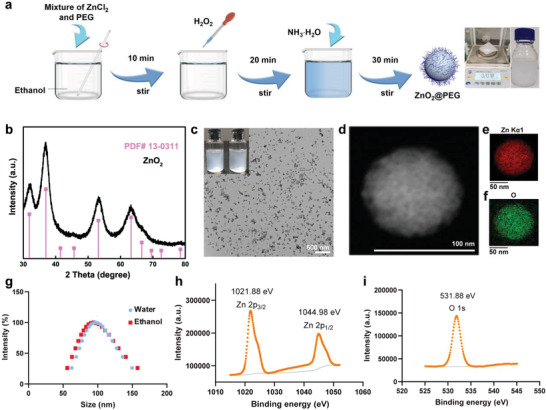
Characterization of ZnO_2_@PEG NPs. a) Simple synthesis procedure of ZnO_2_@PEG NPs (right: weight and picture of ZnO_2_@PEG NPs by adding ten times of reactant amount); b) the XRD pattern of ZnO_2_@PEG NPs; c) TEM images of ZnO_2_@PEG nanoparticles with the insert picture of ZnO_2_ and ZnO_2_@PEG NPs solutions; d–f) HAADF image of ZnO_2_@PEG NPs and corresponding element mapping images; g) DLS results of ZnO_2_@PEG NPs in water and ethanol; h,i) XPS results of Zn 2p and O 1s.

A simple synthesis method can facilitate the nanomaterials synthesis procedures and promote the potential clinic translation.^[^ ^16^ ^]^ We have introduced a simple synthesis method for ZnO_2_@PEG NPs, described in **Figure** **2**a (left), which has the potential for scale‐up (right: weight and solution of synthesized ZnO_2_@PEG NPs with three times magnification of the described procedures). Specifically, 1 mL solution of ZnCl_2_ (2 m) and 1 mL solution of polyethylene glycol‐2000 (PEG) (100 mg mL^−1^) were added into the beaker, which contained 60 mL ethanol. After stirring for 30 min, 200 µL H_2_O_2_ (30%) solution was slowly dropped into the system and further stirred for 30 min. Subsequently, 20 µL NH_3_·H_2_O solution was rapidly injected into the system and reacted for 30 min. The whole solution was centrifugated at 13 000 rpm for 10 min and then washed with water twice. Subsequently, to remove large‐size ZnO_2_@PEG NPs, the solution was centrifugated at 3000 rpm for 5 min, and the upper supernatant was collected. Judging from the X‐ray diffraction (XRD) pattern (Figure 2b), the ZnO_2_ NPs were successfully synthesized. The PEG was introduced to not only control the size of ZnO_2_ NPs but also enhance the biocompatibility, and the Fourier transform infrared spectroscopy (FTIR) spectra verified the existence of PEG (Figure S1, Supporting Information). As Figure 2c depicts, the ZnO_2_@PEG NPs had a size of about 100 nm and presented well dispersibility. The high‐angle annular dark‐field (HAADF) images (Figure 2d) and elements mapping images (Figure 2e,f) and corresponding energy dispersive X‐ray spectrum (Figure S2, Supporting Information) further confirmed the successful synthesis of ZnO_2_@PEG NPs. In addition, the dynamic light scattering (DLS) result (Figure 2g) of ZnO_2_@PEG NPs in ethanol and water both showed the size of about 100 nm, which was in accordance with the transmission electron microscopy (TEM) results. The DLS results were found to be similar in phosphate buffer solution (PBS) and Dulbecco's modified Eagle medium (DMEM) as in water, and well stability in PBS within 3 days was also detected, indicating the good dispersibility and bio‐applications potential of ZnO_2_@PEG NPs (Figure S3a,b, Supporting Information). The zeta potential of ZnO_2_@PEG NPs was also detected as about +50 mV (Figure S3c, Supporting Information) which might support the uptake in cancer cells. Composition and valence state characterizations of ZnO_2_ were then measured using X‐ray photoelectron spectroscopy (XPS) (Figure S4, Supporting Information), the binding energy of 1044.98 eV agreed with Zn 2p_1/2_ and 1021.88 eV agreed with Zn 2p_3/2_, respectively (Figure 2h,i).^[^ ^17^ ^]^ The binding energy of 531.88 eV for element O 1s was higher than 530 eV (the characteristic peak of O 1s in ZnO), indicating the higher valence state of O (O_2_^2−^).^[^ ^18^ ^]^

**Figure 3:**
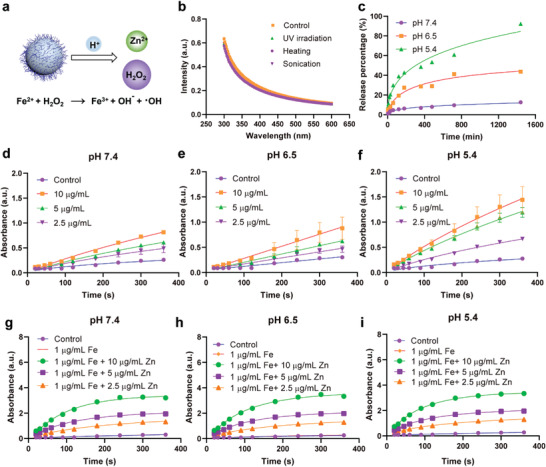
Chemical factory‐like environment‐responsive ability of ZnO_2_@PEG NPs. a) ROS generation process of ZnO_2_@PEG NPs; b) UV‐vis absorbance spectrum of ZnO_2_@PEG NPs under the conditions of UV irradiation, heating, and sonication; c) Zn^2+^ ion release curve of ZnO_2_@PEG NPs (600 µg mL^−1^ Zn in 500 mL solution) at pH 7.4, 6.5, and 5.4, respectively; d–f) TMB (2 mg mL^−1^) oxidation curves of ZnO_2_@PEG NPs at different time points under the conditions of pH 7.4, 6.5, and 5.4, respectively; g–i) TMB (2 mg mL^−1^) oxidation curves of ZnO_2_@PEG NPs with 1 µg mL^−1^ Fe^3+^ at different time points under the conditions of pH 7.4, 6.5, and 5.4, respectively.

Before testing the acidity‐responsive ability of ZnO_2_@PEG NPs (**Figure** **3**a), the other potential external fields including sonification, heat, and UV irradiation were also introduced to evaluate the potential interference. As Figure 3b depicts, no obvious absorbance decrease was detected under the above‐mentioned three external fields stimulation. To assess the acidity‐responsive ability of ZnO_2_@PEG NPs, the Zn^2+^ and H_2_O_2_ release performance was monitored in PBS with various pH values, including pH 7.4, 6.5, and 5.4, respectively. Compared to the low release rate and limited release ratio of the pH 7.4 group (≈13%) within 24 h, the Zn^2+^ ions were released rapidly under acidic conditions. The released amounts were very high, about 44% and 93% for pH 6.5 and pH 5.4 groups, respectively (Figure 3b,c). Then, we utilized the 3,3′,5,5′‐tetramethylbenzidine dihydrochloride (TMB) (2 mg mL^−1^) to further detect the oxidation of ZnO_2_@PEG NPs and hydroxyl radical (•OH) generation efficacy at different pH. Not completely consistent with the Zn^2+^ release curves, the TMB oxidation rate and ratio in the pH 5.4 group were higher than the pH 7.4 and pH 6.5 groups, while seemed similar in the pH 7.4 and pH 6.5 groups (Figure 3d–f), which not only preliminarily provided the foundation for the Fenton/Fenton reactions of ZnO_2_@PEG NPs in liver cancer tissues but also indicated the TMB oxidation ability of ZnO_2_@PEG NPs themselves. In addition, after a 6 min reaction for the ZnO_2_@PEG NPs group and 1 µg mL^−1^ Fe^3+^ ion (FeCl_3_), the TMB had been oxidized almost at the same degree for the pH 7.4, 6.5, and 5.4 groups (Figure 3g,i), which supported the efficient rate of Fenton/Fenton‐like reaction. The efficacy also seemed satisfactory, which provided confidence for the following in vitro and in vivo experiments.

The intracellular levels of ZnO_2_@PEG NPs were measured between the group of cells without any NPs and that were exposed to NPs as previously described.^[^ ^19^ ^]^ Quantification of Zn was accomplished using inductively coupled plasma‐atomic emission spectroscopy (ICP‐AES) of cell lysates. LM3 (hepatocellular carcinoma cell line), RAW264.7 (macrophages), and LO2 (liver cell lines) were plated in 6‐well plates (10^6^ cells per well) and incubated in serum‐free DMEM for 12 h with ZnO_2_@PEG NPs (50 µg mL^−1^ Zn). After incubations, NPs that were not internalized by the cells were washed (three times with PBS) and the cells were centrifuged and resuspended in 200 µL serum‐free medium. Cell counting of each sample was performed with a Countess device (Invitrogen, USA), followed by the addition of an aqueous solution of aqua regia (1 part nitric acid and 3 parts hydrochloric acid by volume). The samples were analyzed by ICP‐AES for the concentration of intracellular levels of Zn. The concentration of Zn was normalized per cell. The estimation of NPs was done based on controlled standard solutions. Compare to the control group, the intracellular Zn contents displayed different levels of increase after incubating with ZnO_2_@PEG NPs (Figure S5, Supporting Information). The highest concentration of Zn was found in LM3 (vs RAW264.7, *p* < 0.01; vs LO2, *p* < 0.01), indicating the higher accumulation of Zn^2+^ in tumor cells after co‐incubation with ZnO_2_@PEG NPs, maybe because that the higher proliferation rate of tumor cells might enhance the uptake and also the increased Zn^2+^ release of ZnO_2_@PEG NPs in the more acidic medium of tumor cells after a long time incubation because of Warburg effect^[^ ^20^ ^]^ (an increase in the rate of glucose uptake and preferential production of lactate) of tumor cells.

**Figure 4:**
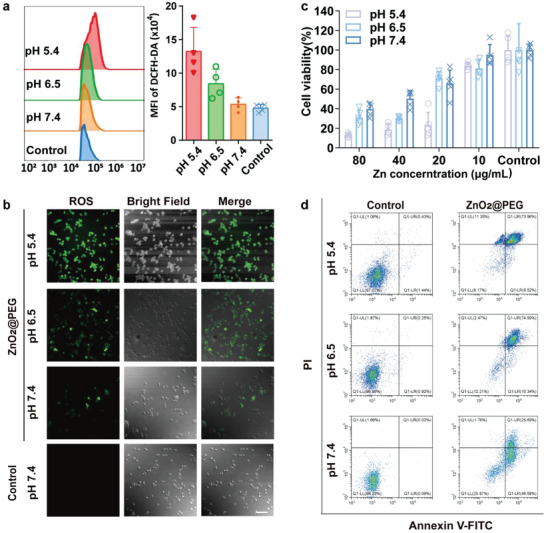
ZnO_2_@PEG NPs treatment generates intracellular ROS and induces cell apoptosis of hepatocellular carcinoma (HCC) in vitro. a) Quantitation of ROS level by using DCFH‐DA probes via flow cytometry (*n* = 4, mean ± SD); b) confocal fluorescence microscope images of LM3 cells for 4 h under different pH (5.4, 6.5, and 7.4), treated with ZnO_2_@PEG NPs (Zn 50 µg mL^−1^). Scale bar = 100 µm; c) cell viability of HCC cells under different pH (5.4, 6.5, and 7.4) after co‐incubation with ZnO_2_@PEG NPs (*n* = 5, mean ± SD); d) contour diagram of Annexin V‐FITC/PI flow cytometry results of LM3 cells after treatment with ZnO_2_@PEG NPs (Zn 100 µg mL^−1^) for 24 h. Q1‐LR (Annexin V‐FITC+/PI−): early apoptotic, Q1‐UR (Annexin V‐FITC+/PI+): late apoptotic, Q1‐LL (Annexin V‐FITC+/PI−): normal cells, Q1‐UL (Annexin V‐FITC−/PI+): necrotic cells. The total amounts of cells in Q1‐LR and Q1‐UR quadrants were determined as apoptotic cells (early apoptotic and late apoptotic cells, respectively). PI, propidium iodide; FITC, fluorescein isothiocyanate.

In addition, the intracellular distribution of ZnO_2_@PEG NPs was observed by confocal laser scanning microscopy (CLSM; Figure S6, Supporting Information). The Cy5‐labeled ZnO_2_@PEG NPs appeared diffusely distributed throughout the cytoplasm and perinuclear regions. Further utilization of Mitotracker‐green probe indicated that most of ZnO_2_@PEG NPs did not co‐localize with mitochondria. To detect the •OH generation in vitro, especially in cancer cells, the dichloro‐dihydro‐fluorescein diacetate (2′,7′‐dichlorofluorescein diacetate (DCFH‐DA)) probe had been introduced on the following groups: control and ZnO_2_@PEG NPs‐treated groups for HCC‐LM3 (human hepatocellular carcinoma cell lines) cells at various pH (7.4, 6.5, and 5.4). Apparently, the fluorescence intensity of ZnO_2_@PEG NPs‐treated cells was much higher than the control group, which could be observed from the flow cytometry results and confocal images (**Figure** **4**a,b). Inspired by the above results, we then assessed the specific anticancer performance of ZnO_2_@PEG NPs group against HCC‐LM3 cells, attributed to the characteristic of low pH, Fe concentration, and catalase activity in the hepatocarcinogenic microenvironment (Figure 4c,d). Cell survival of HCC‐LM3 cells decreased with increasing concentrations of ZnO_2_@PEG NPs, which was more evident in lower pH conditions (Figure 4c). Furthermore, the viabilities of HCC‐LM3 in each group decreased more as the incubation time increased to 54 h (Figure S7, Supporting Information). Besides, we also utilized the live/dead staining method to further evaluate the in vitro anticancer performance of ZnO_2_@PEG NPs. As shown in Figure S8 (Supporting Information), ZnO_2_@PEG NPs presented well anticancer ability under all pH conditions and also exhibited a low pH‐dependent tendency. Note that the cell viability was lower than 30% when the concentration of Zn was 20 µg mL^−1^ at pH 5.4. To explore the mechanism underlying cell death caused by ZnO_2_@PEG nanomaterials, apoptotic cells were quantitatively determined by Annexin V‐FITC and propidium iodide fluorescence staining. The Annexin V‐positive cells were consisted of propidium iodide–negative (early apoptotic) and propidium iodide–positive (late apoptotic) cells, while live cells carry little or no fluorescence. As shown in Figure 4d, negligible apoptosis happened in the three control groups at pH 5.4, 6.5, or 7.4. When incubated with ZnO_2_@PEG NPs, the apoptotic cells and necrotic cells increased significantly with the increasing acidity. The overnight treatment of ZnO_2_@PEG NPs caused the proportion of surviving cells to decrease from over 96% to 10.55 ± 2.13% at pH 5.4, 17.84 ± 10.56% at pH 6.5, 20.57 ± 5.31% at normal pH (Figure S9, Supporting Information). The differences in apoptosis among groups indicated the antitumor effect of ZnO_2_@PEG NPs relied on the release of zinc and H_2_O_2_ under acid conditions. As ZnO_2_@PEG NPs had negligible toxicity on normal liver cells (AML 12 cells) and macrophages (RAW264.7), which resulted from the clearance by catalase in healthy hepatocytes against the slow release of H_2_O_2_ (Figures S10 and S11, Supporting Information). The above in vitro results generally confirmed the specific •OH generation with simultaneous inhibition effect on HCC‐LM3 cells.

### 2.1 ZnO_2_@PEG NPs Hinder the Growth of Liver Cancer by ROS via Fenton Reaction

**Figure 5:**
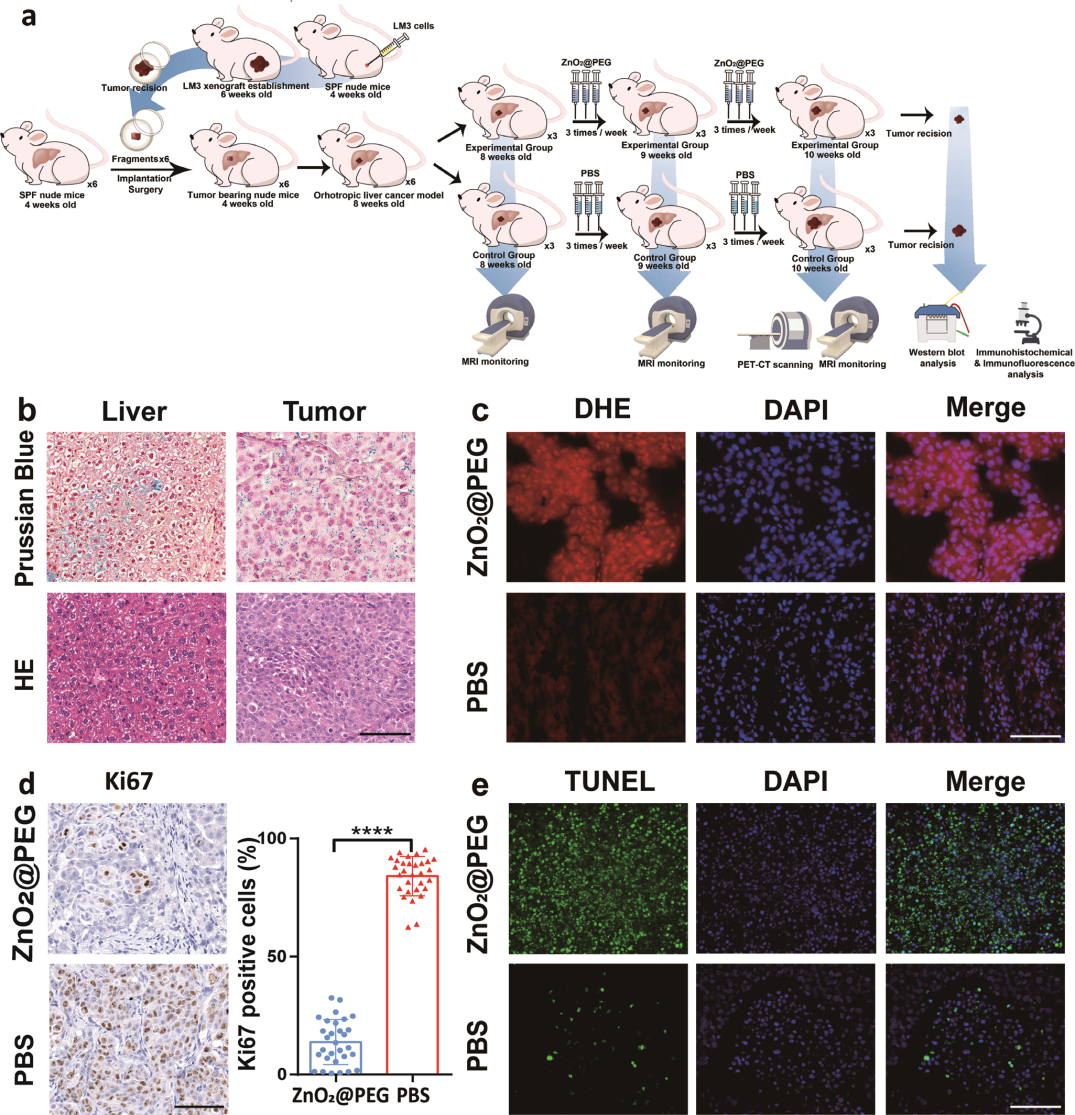
ZnO_2_@PEG NPs treatment generates in situ ROS inside hepatocellular carcinoma (HCC) and induces cell apoptosis of HCC. a) Procedures of establishing orthotopic liver tumor model, conducting the following treatment process and monitoring the treatment efficiency; b) Prussian staining of liver parenchyma and tumor tissue; c) dihydroethidium (DHE) staining for fresh frozen sections of HCC; d) the percentage of Ki67 positive cells in tumor tissue (*n* = 3 × 10, mean ± SD), ****p < 0.0001, Student's *t*‐test (unpaired, two‐tailed); e) TUNEL staining of HCC specimens. Scale bar = 100 µm.

In general, we established the orthotopic liver cancer animal model and monitored the therapy effects in different groups according to the protocol presented in **Figure** **5**a. It is well recognized that iron is crucial in the oxygen transport process, and the liver is the organ with the highest iron storage capacity in the human body.^[^ ^21^ ^]^ In patients with liver cancer, iron is abundant in hepatic parenchyma as well as liver cancer tissues.^[^ ^22^ ^]^ To examine if our tumor‐bearing animal models were consistent with this, we performed Prussian staining on liver parenchyma and tumor tissues from our mice models. Cancerous and noncancerous regions in each group showed scattered excessive iron existence (Figure 5b), enabling the potential of Fenton reaction in hepatocarcinoma background.

Under the condition of tumor acidic microenvironment combined with overloaded iron, accumulated ZnO_2_@PEG NPs in the tumor can generate ROS to damage lipids, DNA, and proteins, inducing obvious necrosis and apoptosis. However, within the alkaline pH environment inside the nontumor liver cell, the Fenton reaction caused by exogenous ZnO_2_@PEG NPs and iron was slow and generated few ROS. At low concentrations, ROS are well‐tolerated by most cells and induce a series of protective responses critical to cell growth, differentiation, and survival.^[^ ^23^ ^]^ Therefore, normal liver cells would not be largely affected by ZnO_2_@PEG NPs. To fully understand the biodistribution and degradation of ZnO_2_@PEG NPs in the body, we first analyzed the blood half‐life of ZnO_2_@PEG NPs by ICP‐AES measurement. The half‐life for ZnO_2_@PEG NPs in the blood was ≈12 min (Figure S12, Supporting Information). Note that the calculated value is semiquantitative, which is difficult to exclude the intrinsic Zn element in the blood. The relatively short circulation time revealed that ZnO_2_@PEG NPs could quickly enter the liver metabolism system and therefore easily be taken by the tumor cell inside the liver, also the enhanced permeability and retention effect could favor the tumor uptake of ZnO_2_@PEG NPs. But we also admitted that the PEG modification alone is not highly satisfactory for long‐term circulation, which encourages us to further investigate the role of surface modifications in nanoparticles metabolism. And therefore, we injected a relatively lower dosage of ZnO_2_@PEG NPs for six times in our experiment to improve the therapeutic effect because it is believed that lower doses and higher frequency may be effective and safer in clinical situation, leading less and controllable side effects. The distributions of ZnO_2_@PEG NPs in major organs were tested by fluorescence imaging and ICP‐AES measurement. Specifically, ZnO_2_@PEG NPs labeled with Cy5 was injected into mice with liver tumor for fluorescence imaging at post 12, 30, and 54 h (Figure S13, Supporting Information). After in vivo fluorescence imaging, the tumor, liver, heart, lung, kidney, and spleen of the mice were collected for ex vivo analysis. The enrichment of ZnO_2_@PEG‐Cy5 NPs was found in the tumor and liver at 12 and 30 h post‐injection. After 54 h, the fluorescence intensity of ZnO_2_@PEG‐Cy5 NPs was mostly localized in the kidney. Tissue Zn content determined by ICP‐AES showed the Zn concentration gradually decreased over 54 h, indicating a low accumulation of ZnO_2_@PEG NPs in all organs and tumor tissue after 54 h (Figure S14, Supporting Information). To investigate ROS production generated by Fenton reaction via ZnO_2_@PEG NPs in vivo, dihydroethidium (DHE) staining of liver and tumor tissue was performed. As shown in Figure 5c and Figure S15 (Supporting Information), the generation of ROS was significantly activated inside the tumor of mice treated with ZnO_2_@PEG NPs, while few ROS were detected in tumor tissue of mice from the control group.

These results above suggested that ZnO_2_@PEG NPs were capable of producing ROS specifically for the tumor in vivo. Overproduced lactate^[^ ^24^ ^]^ with insufficient O_2_ supply^[^ ^25^ ^]^ in the tumor would make it usually present a mild acidic microenvironment.^[^ ^26^ ^]^ As we have observed, tumor regions showed more ROS and more DHE‐positive areas than normal regions, ascribed from the different microenvironment.

As Ki67 protein is present during all active phases of the cell cycle (G1, S, G2, and mitosis) but absent in the resting cell (G0), it is an effective marker for the growth and underlying metastasis. Inhibition of Ki67 expression was confirmed by immunochemical staining in the tumor tissue (Figure 5d). Cell proliferation index represented by ki67 expression was more abundant and extensive in the control group than that of the experimental group. Mice that were administrated with the ZnO_2_@PEG NPs had an almost fourfold reduction in the index than the control group. The mice in the control group had the Ki‐67 proliferation index of 84.07 ± 8.312, while the index of the ZnO_2_@PEG NPs treated group was 13.71 ± 9.511 (*p* < 0.0001) (Figure 5d), indicating the tumor in the control group has been progressing. The apoptosis of cells through ROS was assessed by immunofluorescence for terminal deoxynucleotidyltransferase‐mediated deoxyuridine triphosphate nick end labeling (TUNEL, Figure 5e). The apoptotic nuclei were shown green color when excited by the corresponding wavelength laser under fluorescence microscopy. Cell nuclei were counterstained with 4′,6‐diamidino‐2‐phenylindole (DAPI) and seen as blue. Few TUNEL‐positive cells were detected in the tumor tissues of mice in the control groups, while the TUNEL‐positive cells were widely distributed in tumors of mice treated with ZnO_2_@PEG NPs. The percentage of TUNEL‐positive cells in the ZnO_2_@PEG NPs treated group was higher than that in the control group (*p* < 0.0001) (Figure S16, Supporting Information). Notably, the histomorphological analysis of hematoxylin and eosin (H&E) sections for major organs (brain, heart, lung, spleen, and kidney) did not show any apparent signs of cell damage, necrosis, or inflammation (Figure S17, Supporting Information). Also, peri‐tumor liver sections displayed no hepatocyte necrosis or lipid vacuolation in association with liver damage. Taken together, ZnO_2_@PEG NPs could in situ generate ROS and inhibit tumor growth by inducing tumor cell apoptosis without damaging nontumor tissue. Concerning the long‐term safety of ZnO_2_@PEG NPs, we performed routine blood test, and liver and kidney function tests for all the mice 3 and 60 days after six times injections of ZnO_2_@PEG NPs (Figure S18a, Supporting Information). Nonsignificant abnormalities were found in blood routine test, and liver and kidney function. All the major organs of mice were also examined by the histological examinations by H&E staining, and the results revealed no damage or inflammatory lesion (Figure S18b, Supporting Information).

### 2.2 ZnO_2_@PEG NPs Inhibit the Progression of Liver Cancer In Vivo

**Figure 6:**
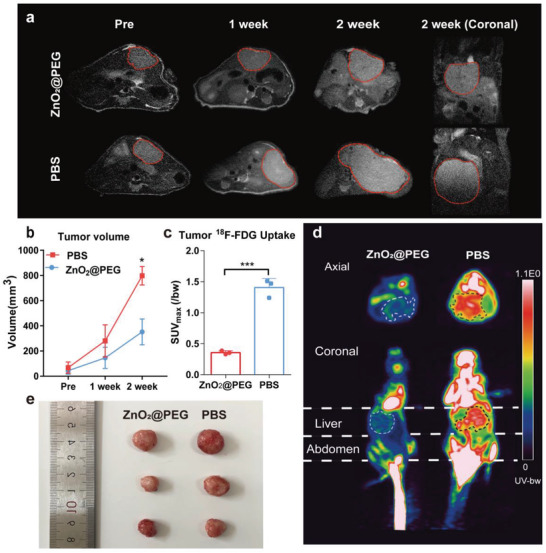
ZnO_2_@PEG NPs treatment blocks the growth of hepatocellular carcinoma (HCC) effectively. a) MRI monitoring in situ tumor growth in the orthotopic HCC nude mice model. The tumors in images are shown by red dashed lines; b) Comparison of tumor volume in the orthotopic HCC nude mice model (*n* = 3, mean ± SD). *p < 0.05 compared to values for the control group at post‐2 week; Student's *t*‐test (unpaired, two‐tailed)); c) SUV_max_ readings of in situ HCC (*n* = 3, mean ± SD). ***p < 0.001, Student's *t*‐test (unpaired, two‐tailed); d) Representative ^18^F‐FDG images of orthotopic HCC tumor‐bearing mice in axial and coronal projection; e) image of isolated tumors.

Tumor response and time to progression have been considered pivotal for treatment assessment of efficacy. With the help of higher‐resolution micro‐magnetic resonance imaging (MRI), liver tumor growth was observed longitudinally at different time points. The orthotopic liver tumors showed hyperintense signal compared with normal tissues on *T*_2_ images from MRI. The series of MRI images for 2 weeks revealed the detectable difference in tumor size between mice with and without ZnO_2_@PEG NPs administration groups (**Figure** **6**a). After 2 weeks treatment, the change of tumor volumes in the two groups was statistically significant (*p* < 0.05), and the tumor volume of the experimental group was only about 44% of that of the control group (Figure 6b,e). Also, the tumor weight of the experimental group was 69% of that of the control group after 2 weeks of treatment (Figure S19, Supporting Information). These data suggested that ZnO_2_@PEG NPs lead to an inhibition of tumorigenesis of liver cancer in vivo. ^18^F‐FDG, the glucose analog, tends to concentrate in the tumor site where metabolize actively. It was used here to make tumors well visualized on positron emission tomography (PET) image, obtained 1 h after ^18^F‐FDG injection. The PET scans (Figure 6c,d) revealed that the tumor SUV_max_ was significantly different between the two groups (*p* < 0.0001). More prominent ^18^F‐FDG in the tumor of control group mice was seen than that in the experiment group (Figure 6d, dashed circle), suggesting that ZnO_2_@PEG NPs lead to remarkable suppression of metabolism and growth of liver cancer. Compared to the control group, lower SUV_max_ readings were observed from mice treated with ZnO_2_@PEG NPs due to larger volume of necrosis inside the tumor.

### 2.3 ZnO_2_@PEG NPs Suppresses Metastasis and Angiogenesis of Liver Cancer

**Figure 7:**
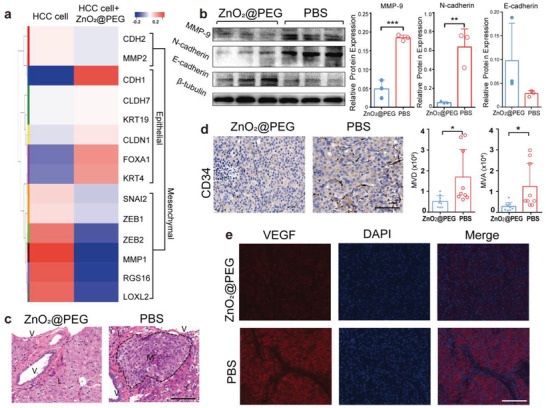
ZnO_2_@PEG NPs treatment effectively abolishes the metastasis and angiogenesis of HCC. a) Heatmap of the expression patterns of EMT‐associated genes in HCC cell. b) Western blot analysis of EMT markers (E‐cadherin, N‐cadherin, MMP‐9) in the resected HCC (*n* = 3, mean ± SD). **p < 0.01, ***p < 0.001 versus the control group treated with PBS, Student's *t*‐test (unpaired, two‐tailed). c) H&E staining of nontumor liver parenchyma of the two groups. The metastasis lesion is delineated by black dashed lines. V = vessel cavity, M = metastasis lesion, L = hepatic parenchyma. Scale bar = 100 µm. d) CD34 immunostaining and the analysis of MVD and MVA in HCC (*n* = 3 × 3, mean ± SD). Scale bar = 100 µm. Data are presented as means ± SD. *p < 0.05, relative to control group; Student's *t*‐test (unpaired, two‐tailed). e) Immunofluorescence staining for VEGF of HCC. Scale bar = 100 µm. Abbreviations: HCC = hepatocellular carcinoma, EMT = epithelial‐mesenchymal transition, MVD = microvessel density, MVA = microvessel area, H&E = hematoxylin and eosin.

Metastasis is the main cause of cancer‐associated death, which mainly hinders treatment success.^[^ ^27^ ^]^ High ^18^F‐FDG uptake is not only a marker for malignancy of liver cancer,^[^ ^28^ ^]^ but also is associated with epithelial‐mesenchymal transition (EMT)‐related proteins^[^ ^29^ ^]^ and consequently a higher frequency of metastatic events. EMT is regulator of the initial steps in the metastatic progression of various tumors.^[^ ^30^ ^]^ Changes in gene expression associated with EMT were summarized in the heatmap (**Figure** **7**a, red: upregulated genes, blue: downregulated genes). When compared with LM3‐HCC cells without treatment, LM3‐HCC cells with ZnO_2_@PEG NPs treatment showed that EMT‐promoting genes presented downregulation and EMT‐suppressing genes showed upregulation. EMT includes several processes that increase the expression of mesenchymal markers such as N‐cadherin and MMP‐9, and concomitant loss of epithelial cell junction proteins such as E‐cadherin.^[^ ^31^ ^]^ Restoring the function of E‐cadherin hinders tumorigenesis and induces tumor cells to become benign phenotype.^[^ ^32^ ^]^ Tumor tissues from the experimental group expressed increased level of E‐cadherin and decreased level of MMP‐9 and N‐cadherin, contrary to that in the control group (Figure 7b). The MMP‐9 and N‐cadherin were overexpressed in liver cancer with PBS injection compared to the group treated with ZnO_2_@PEG NPs, suggesting activation of the EMT pathway in liver cancer without the control of ZnO_2_@PEG NPs, which increased the potency of cell local invasiveness and distant metastasis. Liver cancer holds a high risk of portal vein metastasis, which could strongly damage the liver's regular functions and lead to poor prognosis of patients with liver cancer.^[^ ^33^ ^]^ The histologic results showed an aggressive growth pattern, and the manifestation of which contained intrahepatic metastasis, metastasis tumor in the portal vein, and tumor thrombus (Figure 7c, black dotted area of the right picture). However, in the ZnO_2_@PEG NPs treated group, no obvious metastasis lesion was found outside the primary tumor lesion (Figure 7c, left one).

Liver cancer is prominent for hypervascularity and determined by angiogenesis for tumor growth, supplying a potential target for developing potential therapeutic approaches for liver cancer.^[^ ^34^ ^]^ Sustained tumor growth and metastasis require angiogenesis to facilitate nutrient delivery to the primary tumor and distant spread of tumor cells.^[^ ^35^ ^]^ Given this knowledge and the previous studies suggesting that CD34 is a biomarker of tumor‐associated angiogenesis,^[^ ^36^ ^]^ we evaluated vascular density in tumors to determine whether the reduction in tumor metastasis by treatment resulted from an inadequate blood supply. The positive CD34 stained zone is where neovascular endothelial cells exist and can be used for the determination of tumor microvessel density (MVD) and microvessel area (MVA).^[^ ^37^ ^]^ In Figure 7d, the MVD and MVA of tumor of mice with ZnO_2_@PEG NPs treatment decreased, compared to the control group (*p* < 0.05, *p* < 0.05, respectively). As vascular endothelial growth factor (VEGF) is a direct regulator of tumor‐related angiogenesis,^[^ ^38^ ^]^ we also examined the VEGF protein expression in tumors by immunofluorescence examination. In the control group, intense red fluorescence of intracellular protein expression of VEGF was exhibited in the liver tumors (Figure 7e). Nevertheless, less intense red fluorescence of VEGF expression was observed in the treatment group. The reduction in tumor angiogenesis is highly related to the damage and apoptosis of endothelial cells, mainly due to the increased ROS within the tumor.^[^ ^39^ ^]^ These data suggested that ZnO_2_@PEG NPs lead to remarkable inhibition of metastasis and angiogenesis of liver cancer in vivo.

## 3 Conclusion

In general, we aimed at the unique characteristics of orthotopic liver cancer tissue (including slightly acidic, low catalase activity, the main metabolic site of nanomaterials, the main metabolic site of iron, et al.) to synthesize ZnO_2_@PEG NPs which enable chemical factory‐guaranteed enhanced Fenton/Fenton reactions for cancer therapy. Most of the ZnO_2_@PEG NPs will be passively concentrated in the liver cancer tissues when i.v. injected into mice. In tumor tissues, the ZnO_2_@PEG NPs would rapidly release Zn ion and H_2_O_2_ because of mild acidity and low catalyze activity, subsequently triggering the enhanced Fenton/Fenton‐like reactions with the intrinsic Fe, generating hydroxyl radical for efficient therapy. In contrast, in nontumor liver tissues, the generation of Zn ion and H_2_O_2_ is very slow and will be rapidly degraded into water and oxygen by the intracellular abundant catalase, ensuring the excellent biocompatibility of ZnO_2_@PEG NPs. Moreover, the ZnO_2_@PEG NPs would inhibit the orthotopic liver cancer metastasis to some degree through the regulation of (EMT)‐related proteins expression. Although this research has provided the enlightenment for the combination of CDT with orthotopic liver cancer model because of the super match, there are still other opportunities for matching other nanoparticles, administration methods, or therapy strategies with cancer models. For example, most nanoparticles could be utilized to treat lung‐related diseases including lung cancer via an inhalation approach, and ultra‐small nanoparticles that are mainly concentrated in the kidney could also be introduced to cure kidney‐related diseases including kidney cancer. In addition, when modified with cancer cell membranes, the nanomaterials may have the ability to actively target corresponding cancer models. Overall, it is suggested to first understand the in vivo metabolism of designed nanoparticles, know the specific environment of different cancer models, and then utilized our designed nanoparticles to treat selected cancer models to realize satisfactory treatment. Specifically, there is still room for improvement in this work, and some problems need to be resolved before potential clinical translation. First, the detailed metabolic pathways of the ZnO_2_@PEG NPs in the body should be explored in conjunction with the current advanced imaging methods; the long‐term safety of the ZnO_2_@PEG NPs in the body still needs to be carefully observed; the deep antitumor mechanism of the ZnO_2_@PEG NPs still needs to be further explored; at the same time, the dosage of nanomaterials, the administration interval, and approach need to be optimized. We believe that the combination of our designed nanomaterials and corresponding suitable orthotopic liver cancer model would not only draw the attention of scientists on developing new nanomaterials with diverse properties but also help us to investigate and choose more suitable disease models.

## 4 Experimental Section

### Materials and Reagents

Zinc chloride (ZnCl_2_) (99%), hydrogen peroxide (30%), ammonium hydroxide (25–28%), and polyethylene glycol (PEG‐2000) (Average MW 2000) were all purchased from Sigma‐Aldrich Co., Ltd. Absolute ethanol was obtained from Titan Scientific Co., Ltd. All chemical agents were of analytical grade and were utilized without additional purification. Ultrapure water was obtained using ELGA water purification system (PURELAB Classic) and used during the whole experiments.

### Material Characterizations

XRD measurement was performed on a Rigaku D/MAX‐2250 V X‐ray Diffractometer at Cu K*α* (*λ* = 0.154056 nm) within the 2*θ* range of 30°–80° at a scanning rate of 5 ° min^−1^. TEM images were recorded using JEOL 200CX at 300 kV. In addition, size distribution and zeta potential were monitored with DLS using Malvern Zetasizer (Nano‐ZS90). Agilent 700 Series inductively coupled plasma optical emission spectrometry (ICP‐OES) was employed to quantify element concentration.

### Synthesis of ZnO_2_‐PEG Nanoparticles

The ZnO_2_ nanoparticles were synthesized via a simple coprecipitation method, in which PEG‐2000 was used as the surfactant in the reaction system. First, the A stock solution containing ZnCl_2_ with the concentration of 2 m and B stock solution containing PEG‐2000 with the concentration of 100 mg mL^−1^ were prepared. 1 mL of A and 1 mL of B were added into 100 mL glass flask which contained 60 mL absolute ethanol, and the solution was stirred with the rate of 800 rpm. After stirring for 10 min to generate homogenous solution, 200 µL hydrogen peroxide was rapidly injected into the flask and stirred for further 30 min. Subsequently, 20 µL ammonium hydroxide was dropped into the system for 30 min reaction. Lastly, the solution was centrifugated at the rate of 13 000 rpm for 10 min, then they were washed with pure water. Subsequently, to remove large‐size ZnO_2_@PEG NPs, the solution was centrifugated at 3000 rpm for 5 min, and the upper supernatant was collected. In the contrast, the ZnO_2_ nanoparticles without PEG‐2000 were prepared as the control.

### Zinc (Zn^2+^) Release Performance at Different pH Values

3 mL of ZnO_2_‐PEG solution at Zn concentration of 0.2 mg mL^−1^ was sealed in a dialysis bag by clips (molecular weight cut‐off: 3000 Da.) The dialysis bags were then immersed in the 500 mL beak with 300 mL of buffer media of different pH conditions (7.4, 6.5, and 5.4). The magnesium ion release was conducted in a magnetic stirrer at room temperature with the speed of 100 rpm. At given intervals, 4 mL of the releasing solution was extracted for Zn concentration measurement (unit: µg mL^−1^) using the ICP‐OES.

### Cell Culture and MTT Viability Assays

The human hepatocellular carcinoma (HCC) cell lines LM3,AML 12 (immortalized mouse hepatocyte line), macrophages (RAW264.7)and LO2 (liver cell lines) were obtained from Cell Bank of Chinese Academy of Science (Shanghai, China). HCC‐LM3 cells were maintained in DMEM (Hyclone, USA) supplemented with 10% fetal bovine serum (Gibco, USA), 100 units mL^−1^ penicillin, and 100 µg mL^−1^ streptomycin. HCC‐LM3 cells were seeded into 96‐well plates with a density of 5  ×  10^4^ cells per well for MTT (3‐[4,5‐dimethylthiazol‐2‐yl]‐2,5 diphenyl tetrazolium bromide) assay to measure cell viability. In order to determine whether the ZnO_2_@PEG within acidic environment has impact on cell proliferation, after being cultured for 24 h, the cells were replaced with media of different pH (5.4, 6.5, and 7.4) including ZnO_2_@PEG at Zn concentration of 80, 40, 20, 10 µg mL^−1^ for 24 h. Then 100 µL of 600 µg mL^−1^ MTT reagent was added to each well and incubated in the dark at 37 °C for 4 h, followed by adding 100 µL dimethyl sulfoxide into every well. Finally, cell viability was measured by optical density (OD) at 490 nm using a microplate reader (Bio‐TekELx800, USA).

### Quantification of Cell Apoptosis

To analyze quantitatively the apoptosis of LM3‐HCC cells treated with ZnO_2_@PEG NPs, Annexin V‐FITC‐labeled cells were quantified and considered to be apoptotic cells (*n* = 3 for each group). Briefly, HCC‐LM3 cells were seed in 6‐well plates with a density of 5  ×  10^4^ cells per well. After 24 h of treatment, the samples were washed twice with PBS, re‐suspended in Annexin V binding buffer. 5 µL Annexin V‐FITC and 10 µL PI were used to stain cells for 15 min in the dark at room temperature, according to the manufacturer's protocol for Annexin V‐FITC apoptosis detection kit (Beyotime, China). Annexin V‐FITC and PI‐labeled cells were characterized using flow cytometry (CytoFlex, Beckman CytoFlex, USA), and the data were processed and analyzed using with CytExpert software. The amount of total cells in Q1‐LL quadrant was calculated and summed as the survival ratio.

### Animal Care and HCC Xenograft Mouse Model Establishment

The animal experiments were performed according to the Regional Ethics Committee for Animal Experiments guidelines, and the care regulations authorized by the Administrative Committee of Laboratory Animals of East China Normal University (accreditation number: m+ R20190701). Maximum efforts were made to minimize animals’ pain and suffering, and to reduce the amount of animals needed. The workflow is shown in Figure 4. 6 weeks old nude mice were purchased from Charles River Laboratories (Beijing, China). Mice were housed two per cage in negative pressure isolators and were provided with sterilized food and water ad libitum. All animal procedures were performed under anesthesia with 2% isoflurane inhalation. First, a subcutaneous tumor formation was established by injecting 5 × 10^6^ HCC‐LM3 cells in 50 µL PBS into the lower flank of mice. The animal was monitored every day until the tumor reached a palpable size of 1 cm in all directions. Then the animal was sacrificed, and the flank tumor was resected and cut evenly to small fragments of around 2 mm^3^ with scalpel. Next, other nude mice were anesthetized and opened via subcostal incision. After puncturing into the left liver lobe by a sterile tweezer to form a tunnel beneath the liver capsule, a single fragment was inserted into the newly formed superficial pouch and sealed with surgical glue (Vetbond Tissue Adhesive, 3M, MN, USA), therefore solid tumor deposits established. The abdominal incision was sutured, and the mice were monitored until their recovery from anesthesia. Tumor growth inside mice was monitored by MRI weekly during the whole experiment. After tumor reached a visible size on MRI, the mice were divided into experimental group and control group randomly with similar tumor volume. Mice in experimental group (*n* = 3) were subjected to ZnO_2_@PEG NPs (Zn 2000 µg mL^−1^, 0.2 mL), twice per week for 2 weeks via tail vein injection. The control group (*n* = 3) was injected with 0.2 mL PBS twice per week for 2 weeks. According to the AVMA Guidelines on Euthanasia, if any ulceration or infection occurs in the tumor site, or the existed tumor interfere with eating or free movement, the animals should be euthanized. After euthanasia by CO_2_ was performed on each mouse and liver and tumor tissues were collected for snap‐frozen in liquid nitrogen or fixing in 10% neutral buffered formalin. The obtained snap‐frozen tissues were for protein extraction and ROS determination in vivo. FFPE tissues were sectioned in 5 µm thick sections for histological staining.

### MRI Monitoring

The in vivo monitoring on tumor growth in mice was conducted by a 7.0 T micro‐MRI scanner (Bruker BioSpin, Germany). Each mouse was anesthetized with isoflurane (1.5% in O_2_), positioned supine in the imaging cradle with the liver centered in the coil. Using a *T*_2_‐weighted sequence to measure tumor size (*T*_R_/*T*_E_ = 2000/25.551 ms, field of view 30 × 30 mm, matrix size 256 × 256, slice thickness 0.5 or 0.7 mm), all animals showed a comparable tumor size. Tumor volume was determined by multiplying corresponding slice thickness of *T*_*2*_‐weighted sequence and all tumor areas manually segemented on *T*_*2*_‐weighted images.

### Micro‐PET Imaging using 18F‐FDG

The mice were prewarmed to reach a body temperature of 37 °C before the intravenous administration of 150 µCi of 18F‐FDG (0.6 × 10^−3^ m). The body temperature was maintained at 37 °C during the 1 h uptake period. Micro‐PET imaging was performed with the Inveon micro‐PET‐CT scanner (Siemens Inc., United States). 1 h after 18F‐FDG administration, each mouse was scanned for 10 min. During the micro‐PET imaging, the mice were placed in a supine position inside an imaging chamber, inhaled with isoflurane anesthesia. PET/CT images were processed for reconstruction by the ordered subsets expectation maximization 3D algorithm (OSEM3D), and data were analyzed using the Inveon Research Workplace (IRW) software (Siemens) and processed by PMOD software (version 3.4, PMOD Technologies Ltd., Zurich, Switzerland). The maximum standardized uptake value (SUV_max_) for tumor was measured by drawing a uniform and spherical volume of interest (VOI) upon the tumor zones. The formula of standardized uptake value (SUV) was: [decay‐corrected activity (kBq) per tissue volume (mL)]/ [injected ^18^F‐FDG activity (kBq) per body weight (g)].

### Histology Examination

Dissected liver and tumor tissue were fixed in 4% formaldehyde for 1 day and embedded in paraffin (both Sigma‐Aldrich). Next, the tissue was cut into 0.5 mm^3^ sections by a rotary microtome (Leica Microsystems GmbH, Wetzlar, Germany). Serial sections were cut from the paraffin‐embedded tissues at a thickness of 5 µm for H&E, the Prussian blue, immunohistochemical, immunofluorescence staining, and TUNEL. H&E staining was done with conventional methods using Harris' hematoxylin (Servicebio, Shanghai, China).

*Prussian blue staining*: Prussian blue staining was performed to detect intracellular iron. Briefly, sections were stained with 1:1 mixture of potassium ferrocyanide (Sigma‐Aldrich, USA) and hydrochloric acid. After being washed with distilled water, slides were then counterstained with nuclear fast red solution and washed with distilled water. Finally, the stained sections were finally dehydrated in 95% ethanol, and finally in 100% ethanol.

### Immunohistochemistry

Tissue sections were deparaffinized with xylene, followed by washing with a graded ethanol series (100%, 95%, 85%, and finally 75%). Citrate buffer pH 6.0 (antigen retrieval, Servicebio, Shanghai, China) was used on all sections in a microwave oven, including 10 min moderate heat, 15 min heat preservation, 7 min moderate‐low heat, followed by cooling naturally at room temperature. Then slices were washed with PBS twice (3 min per time) on a decolorizing shaker for 5 min three times. Endogenous peroxidase activity was blocked by using 3% hydrogen peroxide for 25 min in the dark and subsequently washed with PBS on a decolorizing shaker for 5 min for three times, followed by 30 min specimen blocking with 10% bovine serum albumin. Then the slices were incubated with primary antigen at 4 °C overnight. After washing with PBS for three times (5 min per time), slices were incubated with the HRP‐labeled goat anti‐rabbit secondary antibody (1:200, Servicebio, Shanghai, China) for 50 min at room temperature. Sequentially, the color reaction was developed by 3,3‐diaminobenzidine (DAB) and the staining intensity was controlled under observation via microscope. Next, after being counterstained with hematoxylin, differentiation in hydrochloric acid‐ethanol and bluing in ammonia water were performed on all the sections. Finally, the sections were dehydrated in gradient alcohol and xylene, then mounted by neutral gum. Stained sections were observed under a light microscope, and positive cells were stained brown‐yellow or brown in the cytoplasm or nucleus. The following primary antibodies were used: Ki67 (1:200, Servicebio, Shanghai, China), CD34 (1:100, Abcam, MA, USA).

### TUNEL Staining

To observe cell death in situ, the paraffin‐embedded sections were stained using the Roche in situ fluorescein detection kit (Roche, Hertfordshire, UK). Briefly, sections were deparaffinized in xylene, rehydrated in a descending ethanol series, and digested in a protease K working solution for 1 h at 37 °C, before incubated with permeabilization solution for 25 min. After rinsing in PBS, 1:9 mixed solution of terminal deoxytransferase (TdT) and deoxyuridine trisphosphate (dUTP) was applied to serial sections for 3–4 h at 37 °C. Following washed by PBS, the slices were counterstained with DAPI (Servicebio, Hubei, China) for 10 min and dried, followed by sealing with antifluorescence quenching sealing reagents. The percentage of TUNEL positive cells was calculated as the ratio of TUNEL positive cells relative to the amount of the cells (DAPI‐positive nuclei), by particle analysis of ImageJ software.

### Immunofluorescence

### Western Blot Analysis

Tumor tissues from mice were pulverized into powder in liquid nitrogen with radioimmunoprecipitation assay buffer (Beyotime, China), and subsequently supplemented with complete protease inhibitors (Complete EDTA‐free, Roche). The supernatants were collected and the total tissue proteins were extracted herein. The protein concentration was determined by enhanced bicinchoninic acid Protein assay kit (Beyotime Biotechnology, Jiangsu, China). Equal amounts of protein from each group were loaded into sodium dodecyl sulfate‐polyacrylamide gel electrophoresis (Beyotime Institute of Biotechnology, Jiangsu, China) and the protein was transferred onto polyvinylidene fluoride membrane (Millipore, MA, USA). After blocking with 5% non‐fat milk for 2 h, the membranes were incubated overnight with appropriate dilutions of specific primary antibodies at 4 °C. On the next day, the appropriate secondary antibody was incubated for 60 min at room temperature. All the protein bands were visualized using an enhanced chemiluminescence (ECL) chemiluminescent reagent (Millipore, MA, USA) and analyzed by image analysis software (Image Pro Plus 6.0). The following antibodies were used: anti‐E‐cadherin antibody (1:1000; Cell Signaling Technology, MA, USA), anti‐N‐cadherin antibody (1:1000; Cell Signaling Technology, MA, USA), anti‐MMP‐9 antibody (1:1000; Abcam, MA, USA), and *β*‐tubulin(1:5000; Abcam, MA,USA), HRP‐conjugated secondary antibodies (1:1000; Cell Signaling Technology, MA, USA).The intensity of the bands obtained from western blot analysis were quantified using Image‐Pro Plus Quantification 6.0 software.

### RNA Sequencing

Total RNA was extracted from HCC‐LM3 cells treated with or without ZnO_2_@PEG (Zn concentration: 10 µg mL^−1^) for 12 h co‐incubation by using TRIzol reagent (Takara) following the manufacturer's instructions, and immediately frozen in liquid nitrogen. RNA sequencing was performed by Shanghai Majorbio Bio‐Pharm Technology Co., Ltd. using Illumina Miseq system (San Diego, USA). Differential gene expression pattern clustering was performed using Majorbio online platform.

### Determination of ROS Generation

*In vitro quantification*: The intracellular ROS determination was performed by ROS Assay Kit (Beyotime, China) and by flow cytometry (Fortessa flow cytometer, BD Biosciences) according to manufacturer's instruction. Intracellular ROS level was determined by measuring the fluorescence changes, which were resulted from oxidative conversion of cell permeable DCFH‐DA to fluorescent dichlorofluorescein (DCF). HCC‐LM3 cells were seeded in 6‐well plates with a density of 5 × 10^5^ mL^−1^ for 24 h and were incubated with control media at pH 5.4, 6.5, and 7.4 for 6 h in the absence or presence of ZnO_2_@PEG (20 µg mL^−1^ Zn concentration). The cells were washed with serum‐free DMEM solution for three times and incubated with DCFH‐DA for 20 min at 37 °C. Then DCF fluorescence of 10 000 live cells per group was detected by flow cytometer analysis at an excitation wavelength of 488 nm and at an emission wavelength of 525 nm (under FITC channel).

*Intracellular ROS observation*: DCFH‐DA (Beyotime Biotechnologies, Beijing, China) was used as a probe to detect intracellular ROS generation. First, ZnO_2_@PEG NPs were incubated with 50 µg mL^−1^ of Zn at 37 °C for 4 h on confocal dishes. After washed with PBS twice, 10 × 10^−6^ m DCFH‐DA was added to the cell culture medium and then incubated for 30 min at 37 °C. Finally, the stained cells were imaged using a Nikon A1 CLSM.

*In vivo ROS observation*: After being washed with PBS, frozen tumor tissue slices (5 µm thickness) were stained with 1 × 10^−3^ m DHE (Sigma‐Aldrich, MO, USA) for 30 min to detect superoxide existence. After washing with PBS three times (5 min each), the slices were counterstained with DAPI (Servicebio, Hubei, China) for 10 min and washed by PBS three times (5 min each). When subsequent incubation with tissue spontaneous fluorescence quenching agent (Servicebio, Hubei, China) was finished, the slices were dried and sealed with antifluorescence quenching sealing tablets.

### Statistical Analysis

No animals or samples were excluded from analysis due to being outliers during preprocessing of data. Descriptive data were described as means ± the standard deviation (SD).

Student's *t*‐test (unpaired, two‐tailed) was conducted to detect significant differences between data from two groups, and *p* < 0.05 was considered to be statistically significant. Statistical analysis and graphs were processed using GraphPad Prism 7.0 (GraphPad Inc., USA).

*Ki67 Proliferation index*: The proliferation index was determined by calculating the number of Ki67‐positive cells per total number of cells in ten fields at × 40 magnification and expressing it as a percentage.

*Determination of MVD and MVA*: Any CD34^+^ positive‐stained endothelial cell or endothelial cell cluster that was clearly separated from adjacent microvessels, tumor cells, and connective elements was calculated as one microvessel. The mean microvessel count of the five areas with the most intense neovascularization (hot spots) was considered to be the MVD, recorded in terms of the number of vessels per unit area (mm^−2^). MVA was also analyzed by the image analysis software (Image Pro Plus 6.0) and was recorded as the area of vessels per observed area (mm^2^ mm^−2^).

## Conflict of Interest

The authors declare no conflict of interest.

## Supporting information

Supporting Information

## Data Availability

The data that support the findings of this study are available from the corresponding author upon reasonable request.
