# Supplementary material for: Chemical Factory‐Guaranteed Enhanced Chemodynamic Therapy for Orthotopic Liver Cancer
Source: Adv Sci (Weinh). 2022 Jun 16;9(23):2201232. doi: 10.1002/advs.202201232 (PMC9376848; doi:10.1002/advs.202201232)
Supplement: Supplementary file 1 — Supporting Information [file ADVS-9-2201232-s001.pdf]

Supporting Information

Chemical Factory-guaranteed Enhanced Chemodynamic Therapy for Orthotopic Liver Cancer

Zhongmin Tang,‡ Shiman Wu,‡ Peiran Zhao,‡ Han Wang, Dalong Ni, Huiyan Li, Xingwu Jiang, Yelin Wu, Yun Meng, Zhenwei Yao, * Weibo Cai, Wenbo Bu **

Dr. Z. Tang, Prof. Y. Wu, Dr. Y. Meng, Prof. W. Bu
Tongji University Cancer Center, Shanghai Tenth People's Hospital, Tongji University School of Medicine, Shanghai 200072, P. R. China
Email: sk_wuyelin@tongji.edu.cn; wbbu@fudan.edu.cn

Dr. S. Wu, Prof. Z. Yao
Department of Radiology, Huashan Hospital, Fudan University, Shanghai 200040, P. R. China
Email: zwyao@fudan.edu.cn

Dr. P. Zhao, Dr. H. Li, Dr. X. Jiang, Prof. W. Bu
Department of Materials Science and State Key Laboratory of Molecular Engineering of Polymers, Fudan University, 220 Handan Road, Shanghai 200438, P.R. China;

Dr. Z. Tang, Prof. W. Cai
Departments of Radiology, Medical Physics, Materials Science & Engineering, Pharmaceutical Sciences, University of Wisconsin − Madison, Madison, WI 53705, United States

Dr. H. Wang, Prof. D. Ni
Ruijin Hospital, Shanghai Jiao Tong University School of Medicine, Shanghai, 200240, P.R. China

‡These authors contributed equally to this work.

Key words: chemical factory; metabolism; Fenton; liver cancer; zinc peroxide

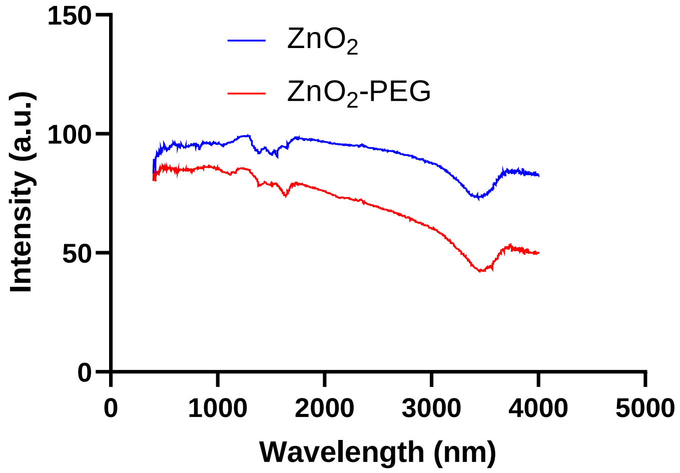

Figure S1. Fourier Transform Infrared Spectroscopy (FTIR) spectra of ZnO_2 and ZnO_2@PEG nanoparticles (NPs).

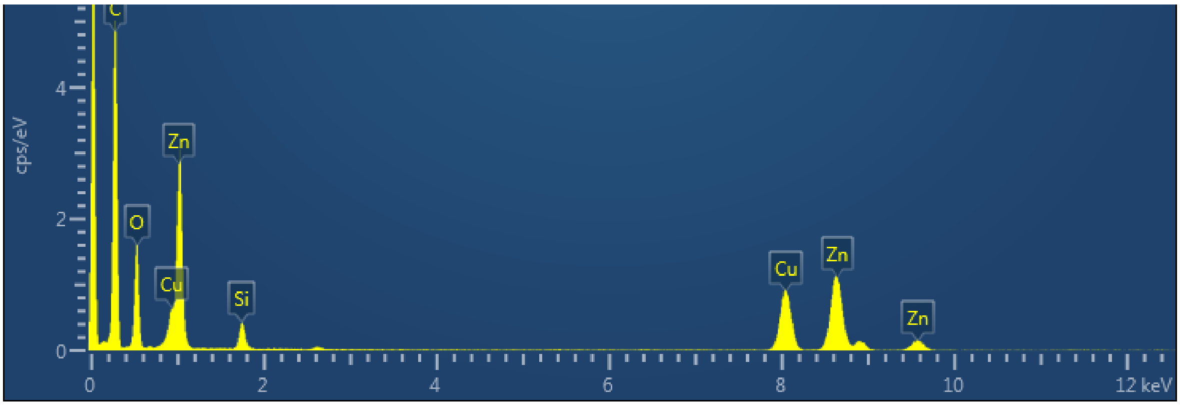

Element	wt%	wt% Sigma
C	57.13	0.34
O	15.31	0.26
Si	2.12	0.07
Cu	11.14	0.15
Zn	14.31	0.18

Figure S2. Energy Dispersive X-ray (EDX) spectrum of ZnO_2@PEG NPs and its quantitative result.

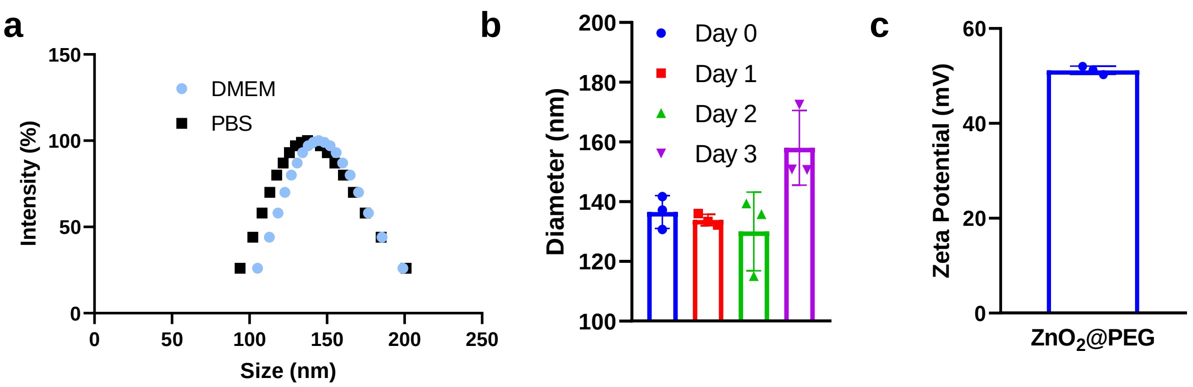

Figure S3. (a) DLS results of ZnO_2@PEG NPs in PBS and DMEM. (b) DLS results of ZnO_2@PEG NPs in

PBS within three days. (c) Zeta potential of ZnO_2@PEG NPs.

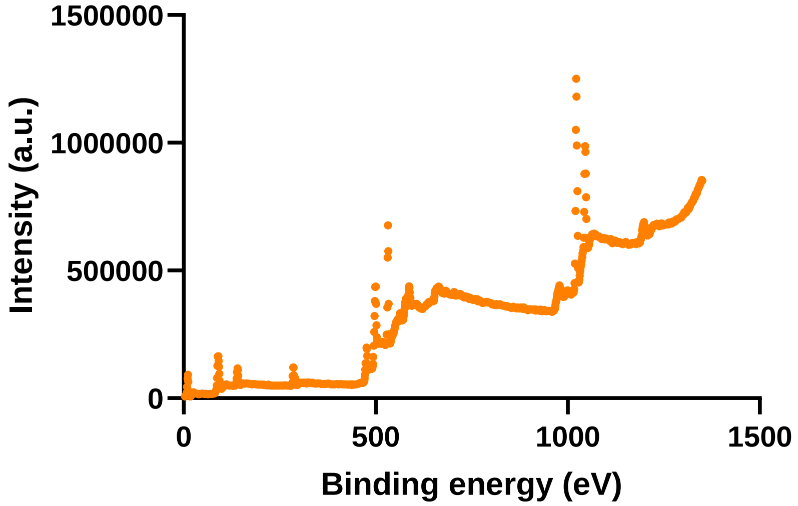

Figure S4. XPS full spectrum of ZnO_2@PEG NPs.

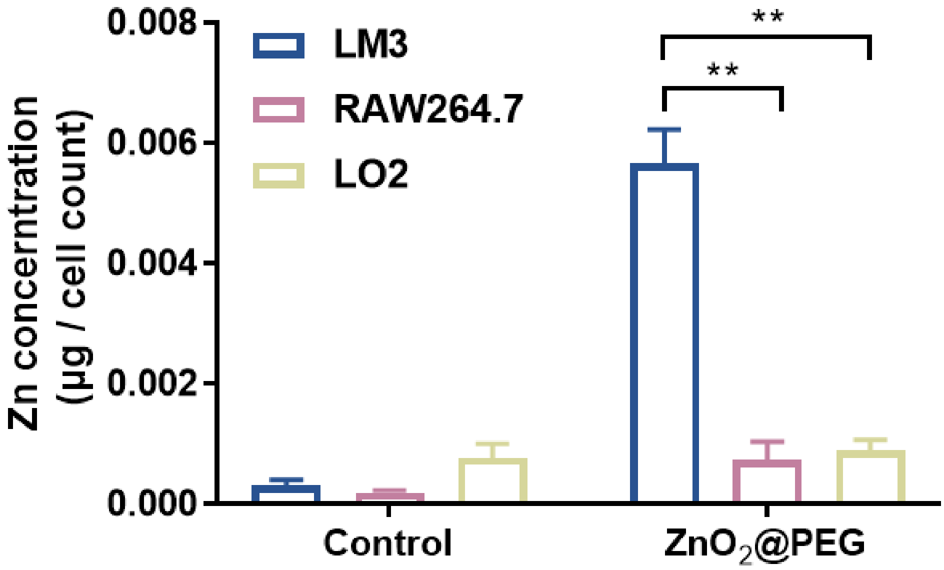

Figure S5. The uptake of NPs in LM3 (hepatocellular carcinoma cell line), RAW264.7 (macrophages) and LO2 (liver cell lines) was measured by ICP-MS using Zn levels, in comparison of control group (n=3, mean± SEM). ** P<0.01, Student *t* test (unpaired, two-tailed).

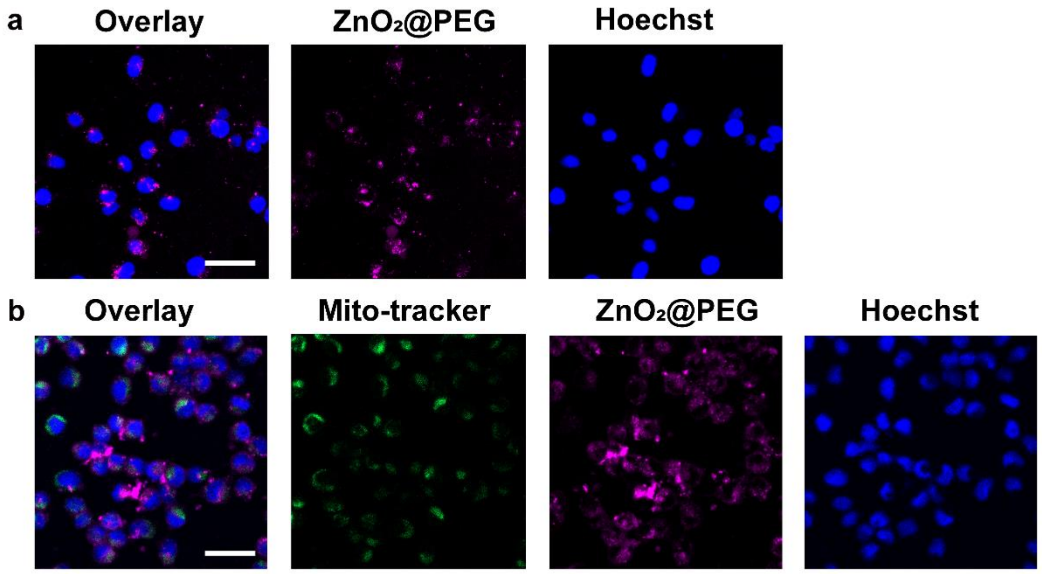

Figure S6. The intracellular distribution of ZnO_2@PEG-Cy5 NPs (a) and with MitoTracker stained mitochondria (b) via CLSM. Scale bar=100 μm. As ZnO_2@PEG-Cy5 NPs was localized in cytoplasm and around the nucleus, without a clear distinction between nuclear and cytoplasmic regions, MitoTracker staining was performed to determine if ZnO_2@PEG-Cy5 NPs would be colocalized with mitochondria. Staining with Mitotracker-green probe indicated that most of ZnO_2@PEG NPs did not co-localize with mitochondria.

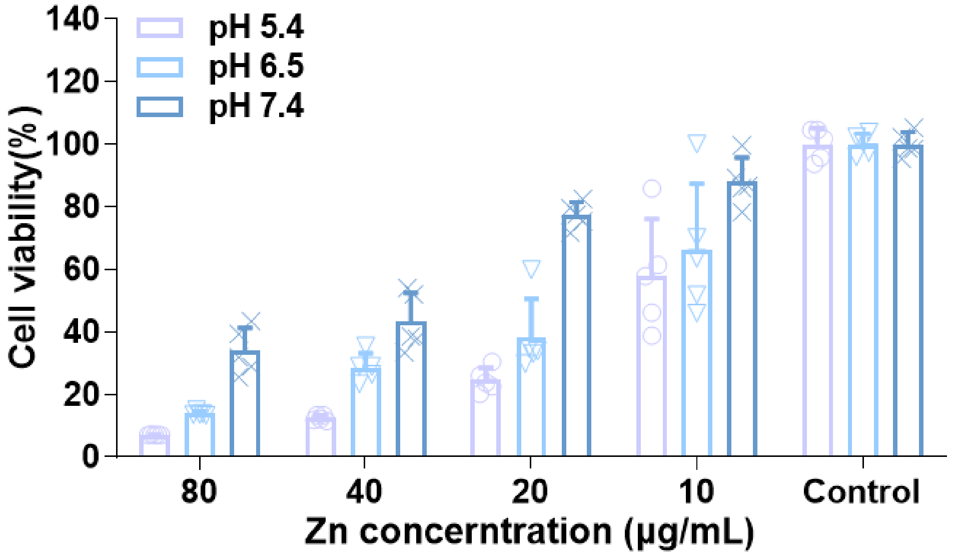

Figure S7. Cell viability of HCC cells under different pH (5.4, 6.5 and 7.4) after co-incubation with ZnO_2@PEG NPs (n=5, mean ± SD).

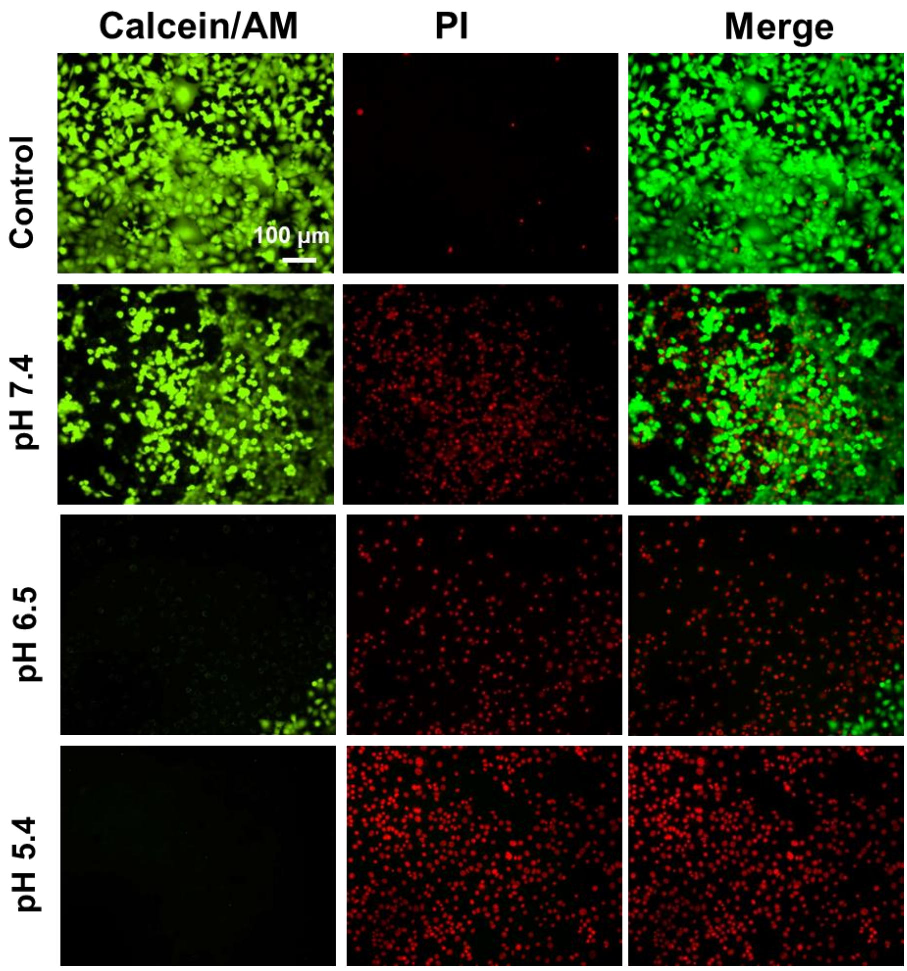

Figure S8. Confocal images of HCC-LM3 cells after co-incubation with ZnO_2@PEG NPs under different conditions (dead cells: Red, live cells: Green).

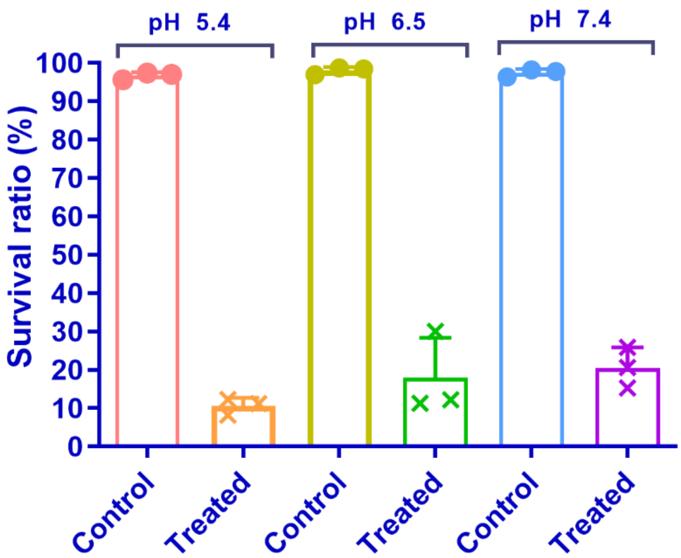

Figure S9. The percentage of viable cells (n=3, mean ± SD) was recorded from the Annexin V-FITC negative and PI negative zone. “Treated” represented the cells treated by ZnO_2@PEG NPs.

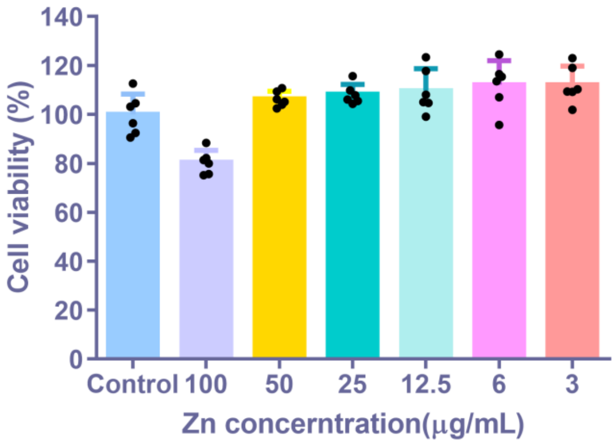

Figure S10. Cytotoxicity assessment of ZnO_2@PEG NPs. Cell viability (n=6, mean ± SD) of AML 12 (immortalized mouse hepatocyte line) after 24 h incubation with ZnO_2@PEG NPs at the Zn concentration 3, 6, 12.5, 25, 50,100 μg/mL. ZnO_2@PEG NPs did not obviously inhibit the viability of cells in the range of concentrations used.

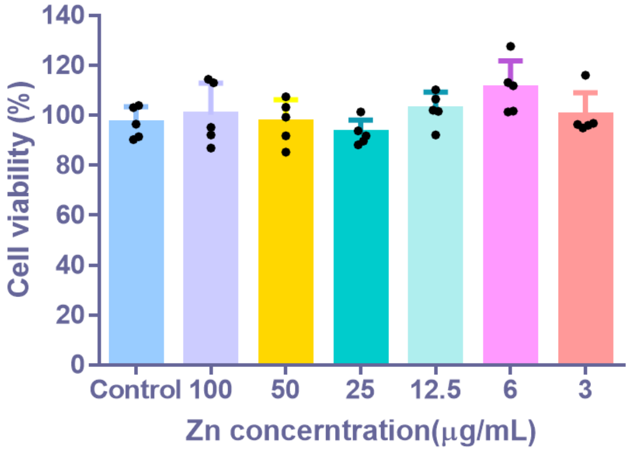

Figure S11. Cytotoxicity assessment of ZnO_2@PEG. Cell viability (n=5, mean ± SD) of RAW264.7 cells after 24 h incubation with ZnO_2@PEG NPs at the Zn concentration 3, 6, 12.5, 25, 50,100 μg/ mL. ZnO_2@PEG NPs did not obviously inhibit the viability of RAW264.7 cells in the range of concentrations used.

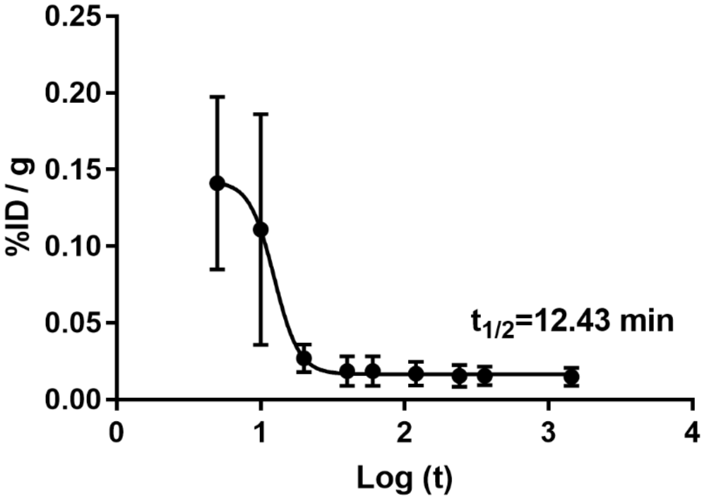

Figure S12. The blood half-life of ZnO_2@PEG NPs (n=3, mean ± SD). Note that the calculated value is semiquantitative, which is difficult to exclude the intrinsic Zn element in the blood. The relatively short circulation time revealed that ZnO_2@PEG NPs could quickly enter in the liver metabolism system and therefore easily be taken by the tumor cell inside liver. But we also admit that the PEG modification alone is not highly satisfactory for long-term circulation, which encourages us to further investigate the role of surface modifications in nanoparticles metabolism. And therefore we injected relatively lower dosage of ZnO_2@PEG NPs for six times in our experiment to improve the therapeutic effect because it is believe that lower doses and higher frequency may be effective, safer in clinical situation, leading less and controllable side effects.

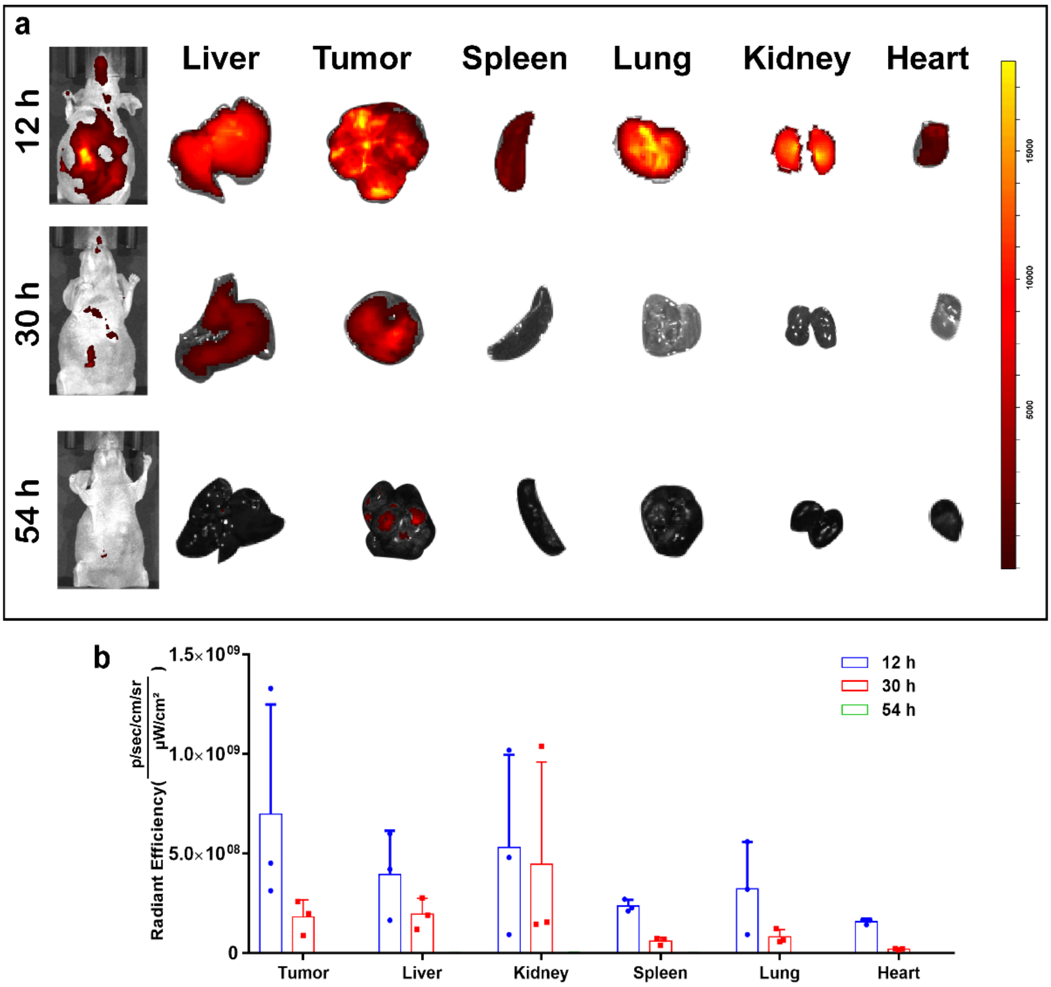

Figure S13. (a) Fluorescent images of nude mice with liver tumor and harvested tissues. (b) Quantitative analyses of fluorescent intensity in tumor and major organs collected from the mice treated with ZnO_2@PEG NPs after 12, 30, 54 h (n=3, mean ± SD).

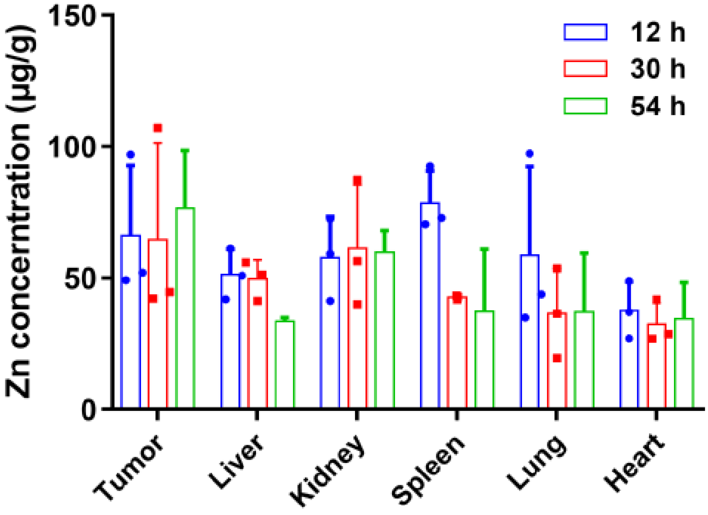

Figure S14. Quantitative analyses of Zn content in major organs collected from the mice treated with ZnO_2@PEG NPs after 12, 30, 54 h (n=3, mean ± SD).

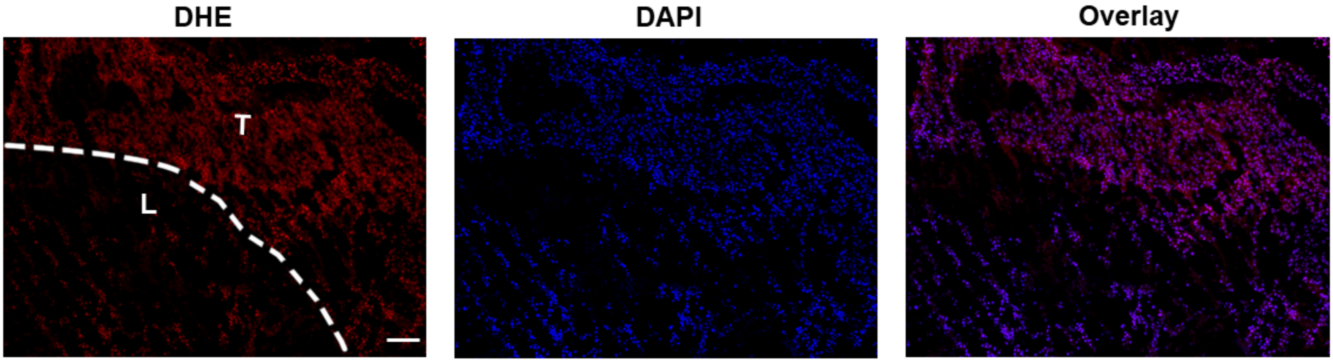

Figure S15. DHE staining images of HCC tumor tissue (T) and tumor-adjacent liver tissue (L) of ZnO_2@PEG NPs-injected group. Scale bar=100 µm.

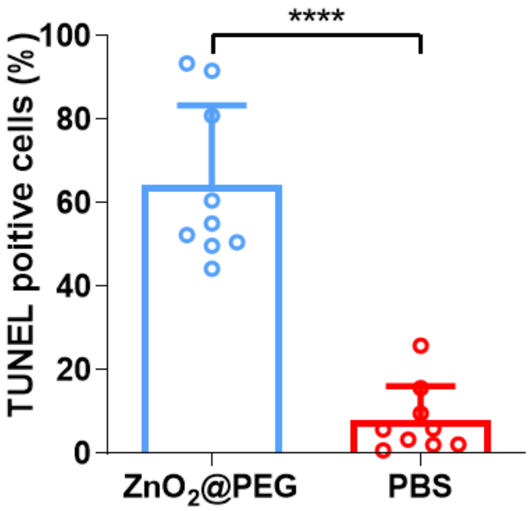

Figure S16. The percentage of TUNEL positive cells in tumor tissue of the ZnO_2@PEG NPs-treated group and the PBS-treated group. (n=3×3, mean ± SD). ZnO_2@PEG NPs-treated group: 64.32±19.07, PBS-treated group: 7.95±8.15, ****P<0.0001; Student *t* test (unpaired, two-tailed).

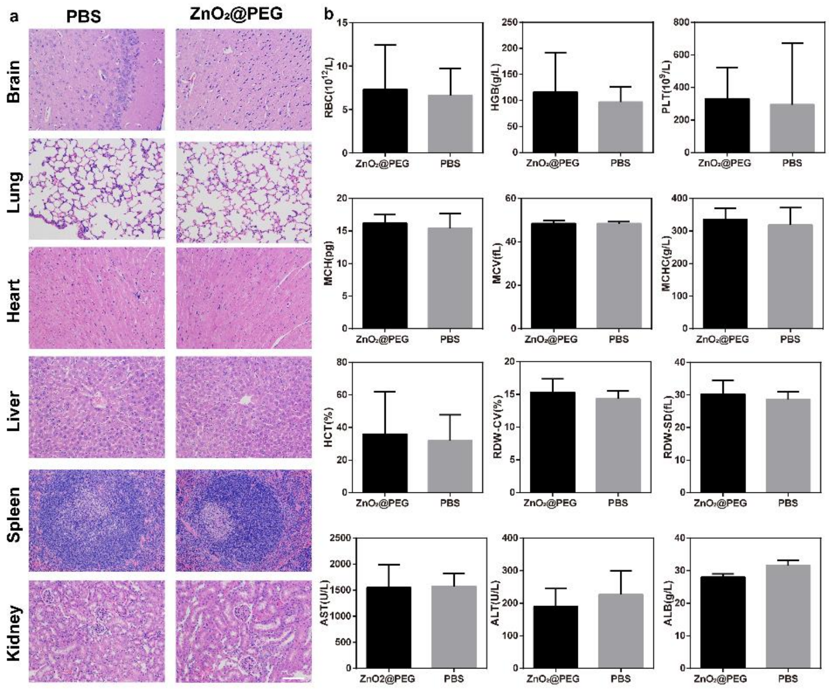

Figure S17. *In vivo* toxicity evaluation of ZnO_2@PEG NPs. (a) Blood regular test, biochemistry index (n=3, mean ± SD); (b) HE-stained tissues (200x) of heart, liver, spleen, lung, and kidney of nude mice with hepatocellular carcinoma. No differences in blood tests in ZnO_2@PEG NPs-injected group compared to PBS control group. The cell morphology and size of nucleus did not differ between two groups, supporting biocompatibility of ZnO_2@PEG NPs. Scale bar=100 μm.

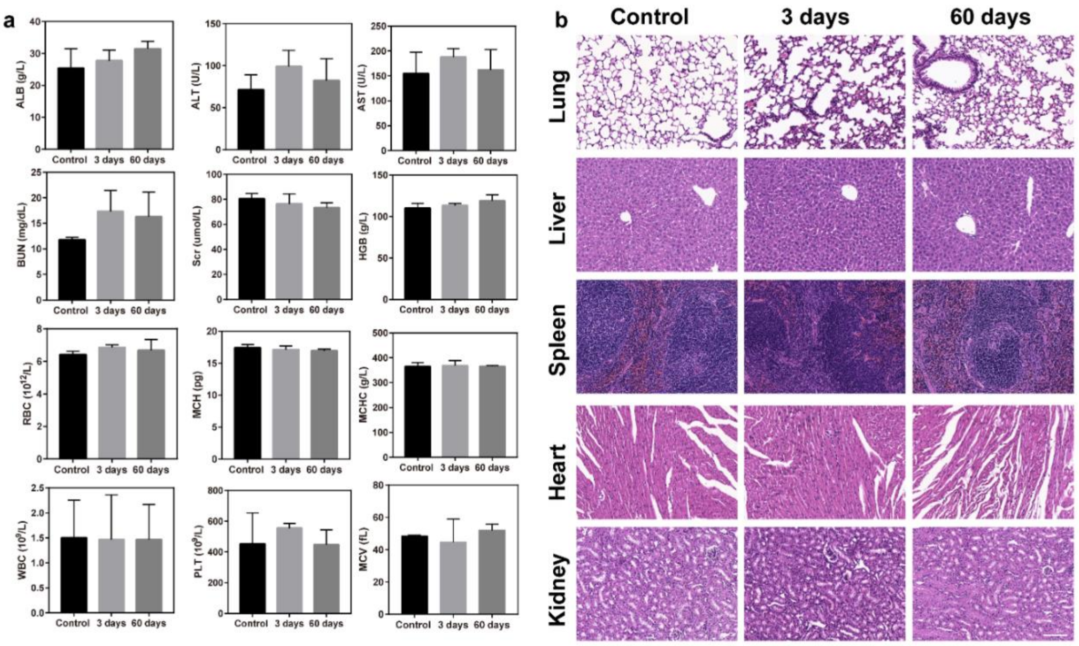

Figure S18. (a) Routine blood test, liver and kidney function test results of mice injected with PBS, mice injected with ZnO_2@PEG NPs (3-day observation), mice injected with ZnO_2@PEG NPs (60-day observation) (n=3, mean ± SD). (b) H&E staining of major organs of mice injected with PBS, mice injected with ZnO_2@PEG NPs (3-day observation), mice injected with ZnO_2@PEG NPs (60-day observation). Scale bar=100 μm.

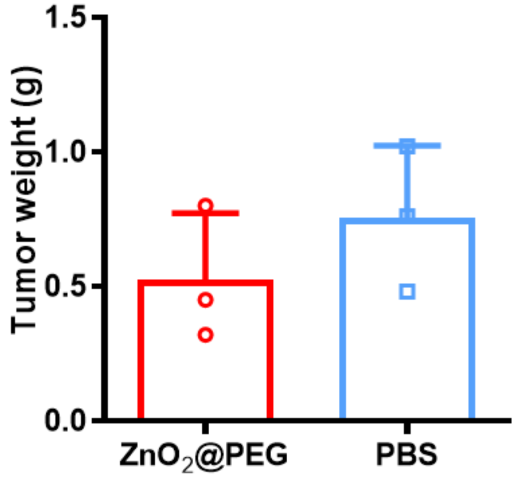

Figure S19. Comparison for mean tumor weight between the ZnO_2@PEG NPs-treated group and the PBS-treated group. The mean tumor weight of the ZnO_2@PEG NPs-treated group was 69% of that of control group after 2-week treatment (n=3, mean ± SD).
